# Sheffield General Infirmary

**Published:** 1892-11-19

**Authors:** 


### HOSPITAL CONSTRUCTION.

### SHEFFIELD GENERAL INFIRMARY.

In reference to the article of our Special Commissioner on this institution, which appeared on p. 109 of the number for November 12th, we are glad to learn on authority that there were special circumstances which accounted for the fact that the offices of the new building were not in such good order as the remainder of the hospital. Having regard to the efficiency of the whole institution we have no doubt that these special circumstances, including the absence of the Sister-in-Charge on her holiday, sufficiently accounted for the temporary irregularity.

To fulfil any useful purpose, our Commissioner must report exactly what he finds at the time of his visit, and for this reason a series of reports from him published in these columns constitute the most telling justification of the voluntary system of hospital management which has ever been published.

We have always determinedly advocated an increase in the rate of remuneration paid to nurses in responsible positions, by hospital committees. In pursuance of this policy, our Commissioner recommended larger salaries at Sheffield, because the work done warrants such an increase of remuneration. In these circumstances it would be curious, indeed, were such an advocacy to be taken as a reflection upon the way in which nurses discharge their duty. Good work demands good payment, and if ever there was good work done by a nursing staff, it is to be met with at the Sheffield Infirmary.

